# Implication of EZH2 in the Pro-Proliferative and Apoptosis-Resistant Phenotype of Pulmonary Artery Smooth Muscle Cells in PAH: A Transcriptomic and Proteomic Approach

**DOI:** 10.3390/ijms22062957

**Published:** 2021-03-15

**Authors:** Karima Habbout, Junichi Omura, Charifa Awada, Alice Bourgeois, Yann Grobs, Vinod Krishna, Sandra Breuils-Bonnet, Eve Tremblay, Ghada Mkannez, Sandra Martineau, Valérie Nadeau, Florence Roux-Dalvai, Mark Orcholski, Jey Jeyaseelan, David Gutstein, François Potus, Steeve Provencher, Sébastien Bonnet, Roxane Paulin, Olivier Boucherat

**Affiliations:** 1 Pulmonary Hypertension Research Group, Centre de Recherche de l’Institut Universitaire de Cardiologie et de Pneumologie de Québec, Québec City, QC G1V 4G5, Canada; karima.habbout.1@ulaval.ca (K.H.); junp0103@gmail.com (J.O.); charifa.awada.1@ulaval.ca (C.A.); alice.bourgeois.2@ulaval.ca (A.B.); yann.grobs.1@ulaval.ca (Y.G.); sandra.breuils-bonnet@criucpq.ulaval.ca (S.B.-B.); eve.tremblay@criucpq.ulaval.ca (E.T.); ghada.mkannez.1@ulaval.ca (G.M.); sandra.martineau@criucpq.ulaval.ca (S.M.); valerie.nadeau@criucpq.ulaval.ca (V.N.); mark.orcholski@criucpq.ulaval.ca (M.O.); francois.potus@criucpq.ulaval.ca (F.P.); Steeve.Provencher@criucpq.ulaval.ca (S.P.); Sebastien.Bonnet@criucpq.ulaval.ca (S.B.); roxane.paulin@criucpq.ulaval.ca (R.P.); 2 Janssen Research & Development, Spring House, PA 19477, USA; vkrish10@ITS.JNJ.com (V.K.); jjeyasee@ITS.JNJ.com (J.J.); David.Gutstein@nyulangone.org (D.G.); 3 Proteomics Platform, CHU de Quebec, Université Laval Research Center, Quebec City, QC G1V 4G2, Canada; Florence.Roux-Dalvai@crchudequebec.ulaval.ca; 4 Department of Medicine, Université Laval, Québec City, QC G1V 4G5, Canada

**Keywords:** vascular remodeling, epigenetics, metabolism, right ventricle

## Abstract

Pulmonary arterial hypertension (PAH) is a progressive disorder characterized by a sustained elevation of pulmonary artery (PA) pressure, right ventricular failure, and premature death. Enhanced proliferation and resistance to apoptosis (as seen in cancer cells) of PA smooth muscle cells (PASMCs) is a major pathological hallmark contributing to pulmonary vascular remodeling in PAH, for which current therapies have only limited effects. Emerging evidence points toward a critical role for Enhancer of Zeste Homolog 2 (EZH2) in cancer cell proliferation and survival. However, its role in PAH remains largely unknown. The aim of this study was to determine whether EZH2 represents a new factor critically involved in the abnormal phenotype of PAH-PASMCs. We found that EZH2 is overexpressed in human lung tissues and isolated PASMCs from PAH patients compared to controls as well as in two animal models mimicking the disease. Through loss- and gain-of-function approaches, we showed that EZH2 promotes PAH-PASMC proliferation and survival. By combining quantitative transcriptomic and proteomic approaches in PAH-PASMCs subjected or not to EZH2 knockdown, we found that inhibition of EZH2 downregulates many factors involved in cell-cycle progression, including E2F targets, and contributes to maintain energy production. Notably, we found that EZH2 promotes expression of several nuclear-encoded components of the mitochondrial translation machinery and tricarboxylic acid cycle genes. Overall, this study provides evidence that, by overexpressing EZH2, PAH-PASMCs remove the physiological breaks that normally restrain their proliferation and susceptibility to apoptosis and suggests that EZH2 or downstream factors may serve as therapeutic targets to combat pulmonary vascular remodeling.

## 1. Introduction

Pulmonary arterial hypertension (PAH) is a progressive and fatal disease defined by a mean pulmonary artery (PA) pressure at rest greater than 20 mmHg. Pathological changes in this disease involve vasoconstriction and remodeling of distal PAs, leading to increased vascular resistance, right ventricular failure, and premature death [1,2]. Despite major effort to understand the complex and interrelated pathways underlying PAH, current available drugs that primarily address the vasoconstrictive phenotype fail to substantially improve patient outcomes, thus necessitating redirection of therapeutic strategies [3,4].

Excessive proliferation and resistance to apoptosis of PA smooth muscle cells (PASMCs), as observed in cancer cells, are considered as critical contributors to vascular remodeling in PAH [1,2]. A complex web of alterations that converge towards the acquisition and maintenance of this abnormal phenotype has been documented. Among these, PAH-PASMCs undergo improper mitochondrial dynamics coupled with a multifaceted metabolic reprogramming with a shift from oxidative phosphorylation to aerobic glycolysis [5] accompanied by increased glutaminolysis [6]. Alongside this bioenergetic adaptation to fulfill their biosynthetic demands during proliferation, PAH-PASMCs have developed different strategies to tolerate stress and promote their survival, such as enhanced DNA repair capacity and autophagy [1], pinpointing the complex mechanisms underlying PAH.

Over the last few years, epigenetic alterations with functional impacts on gene expression such as DNA methylation, histone modifications, and noncoding RNAs have gained significant interest as drivers of pulmonary vascular remodeling [7]. The influence of epigenetic dysregulation in the abnormal PAH-PASMC behavior is perhaps best exemplified by in vitro data showing that PAH cells isolated from PAH patients exhibit a persistently hyperproliferative, apoptosis-resistant, and proinflammatory phenotype over several passages [8]. Owing to the ability of one single epigenetic modifier to regulate the expression of multiple genes involved in distinct cellular pathways affected, epigenetic modifiers are considered to be attractive therapeutic targets in complex diseases. Nevertheless, although epigenetic modifications are increasingly appreciated as an important contributing factor to PAH, the nature of epigenetic modifier enzymes crucially involved in disease development and progression remains largely unknown.

Enhancer of Zeste Homolog 2 (EZH2), the catalytic component of the polycomb repressive complex 2 (PRC2), is responsible for H3 lysine 27 trimethylation (H3K27me3), a chromatin mark associated with gene repression [9]. EZH2 was documented to be overexpressed in a wide range of cancer types, including lung, liver, prostate, and breast cancers [9]. By inhibiting the expression of tumor suppressor genes, EZH2 was shown to govern the acquisition of a prosurvival and pro-proliferative phenotype providing rationale for PRC2 inhibition as a novel antineoplastic strategy. However, mounting evidence indicates that EZH2-mediated H3K27 trimethylation and subsequent gene silencing is not sufficient to account for all functions of EZH2 in cancer. Indeed, besides its PRC2-dependent methylation function, EZH2 was also documented in cancer cells to physically interact with various proteins to positively regulate their protein stability [10] as well as to cooperate with several transcription factors to stimulate gene transcription [11,12,13,14,15]. Thus, EZH2 appears to rely on multiple cell context-dependent mechanisms to facilitate neoplastic transformation and sustain tumor growth. Although EZH2 was previously shown to be implicated in right ventricular dysfunction [16], its role in pulmonary vascular remodeling process in the setting of PAH remains largely unknown.

In the present study, we demonstrate that EZH2 is overexpressed in PASMCs from PAH patients and animal models mimicking the disease enhancing their proliferation and resistance to apoptosis. By combining transcriptome and proteome profiling in PAH-PASMCs subjected or not to EZH2 knockdown, we found that EZH2 positively regulates a large array of factors critically involved in cell-cycle progression, mitochondrial translation, and tricarboxylic acid (TCA) cycle. Accordingly, we found that molecular inhibition of EZH2 in PAH-PASMCs compromises mitochondrial respiratory capacity. Our study therefore uncovered a critical function of EZH2 in pulmonary vascular remodeling associated with PAH.

## 2. Results

### 2.1. EZH2 Levels in Human PAH and Experimental Models

**Figure 1:**
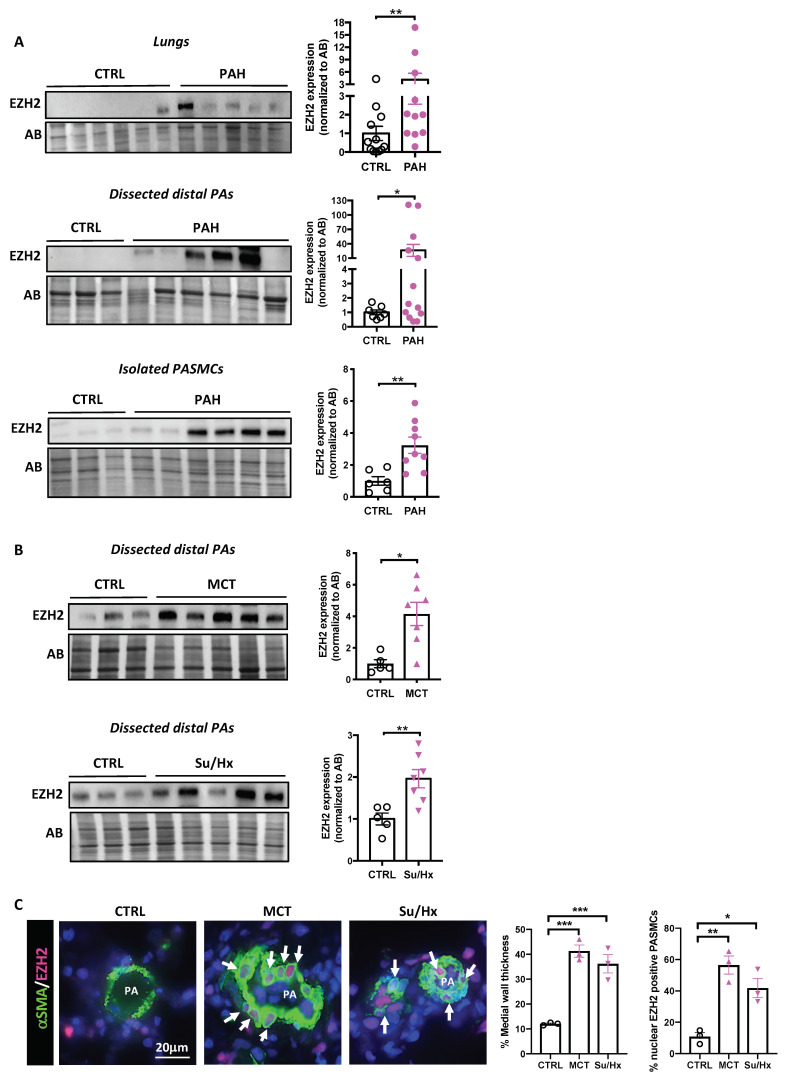
Expression of EZH2 in patients with pulmonary arterial hypertension (PAH) and animal models. (**A**) Representative Western blots and corresponding densitometric analyses of EZH2 expression in lung biopsies, dissected pulmonary arteries (PA), and isolated PA smooth muscle cells (PASMCs) from control (*n* = 6–12) and PAH (*n* = 9–13) patients; (**B**) Representative Western blots and corresponding densitometric analyses of EZH2 expression in PAs dissected from control rats as well as monocrotaline (MCT)- and Sugen/hypoxia (Su/Hx)-induced pulmonary hypertension rat models (*n* = 5–7 rats per group); (**C**) Double immunofluorescence staining for αSMA (green) and EZH2 (red) and DAPI nuclear staining showing nuclear expression of EZH2 in remodeled distal PAs after MCT injury or Sugen/hypoxia exposure compared to nontreated rats. Graphs on the right represent the quantification of the medial wall thickness of distal PAs and the percentage of PASMCs positive for EZH2 in distal pulmonary vessels (*n* = 3 per group, mean of 15 vessels/rat). Arrows mark positive cells. Scale bar = 20 μm. Protein expression was normalized to Amido black (AB). Data are presented as mean ± SEM; * *p* < 0.05; ** *p* < 0.01; and *** *p* < 0.001.

To determine whether EZH2 is implicated in the obliterative vasculopathy that characterized PAH, we first measured its protein expression level in lungs, dissected PAs (<1000 µm in diameter), and isolated PASMCs from control and PAH patients. Regardless of the anatomical level, increased expression of EZH2 was observed in PAH patients (Figure 1A). We next evaluated whether similar changes also occur in animal models recapitulating PAH. To this end, dissected PAs from monocrotaline (MCT)- and Sugen/hypoxia (Su/Hx)-treated rats were used. As observed in human, EZH2 expression was significantly upregulated in MCT- and Su/Hx-challenged rats compared to their normal counterparts (Figure 1B). In agreement with this, marked nuclear localization of EZH2 expression was detected in PASMCs from rat remodeled distal PAs (<75 µm in diameter) identified by co-staining with µSMA, while the signal of EZH2 was barely detectable in normotensive rat PAs (Figure 1C). To complement our approach, we measured EZH2 expression in PA endothelial cells (PAECs) as well as in muscle (quadriceps), liver, and kidney biopsies from control and PAH patients. Contrary to PASMCs, EZH2 expression was unchanged between control and PAH-PAECs (Figure S1). Furthermore, no signal was detected in quadriceps muscle (data not shown), and no major change was seen in kidney and liver (Figure S1). These data, combined with our previous finding showing that EZH2 is augmented in human compensated right ventricle (RV) but markedly decreased in decompensated RV from PAH patients [16], indicate that altered expression of EZH2 in PAH appears to be mainly restricted to the cardiopulmonary system.

### 2.2. Effects of EZH2 Loss- and Gain-of-Function Approaches on PASMC Proliferation and Resistance to Apoptosis

**Figure 2:**
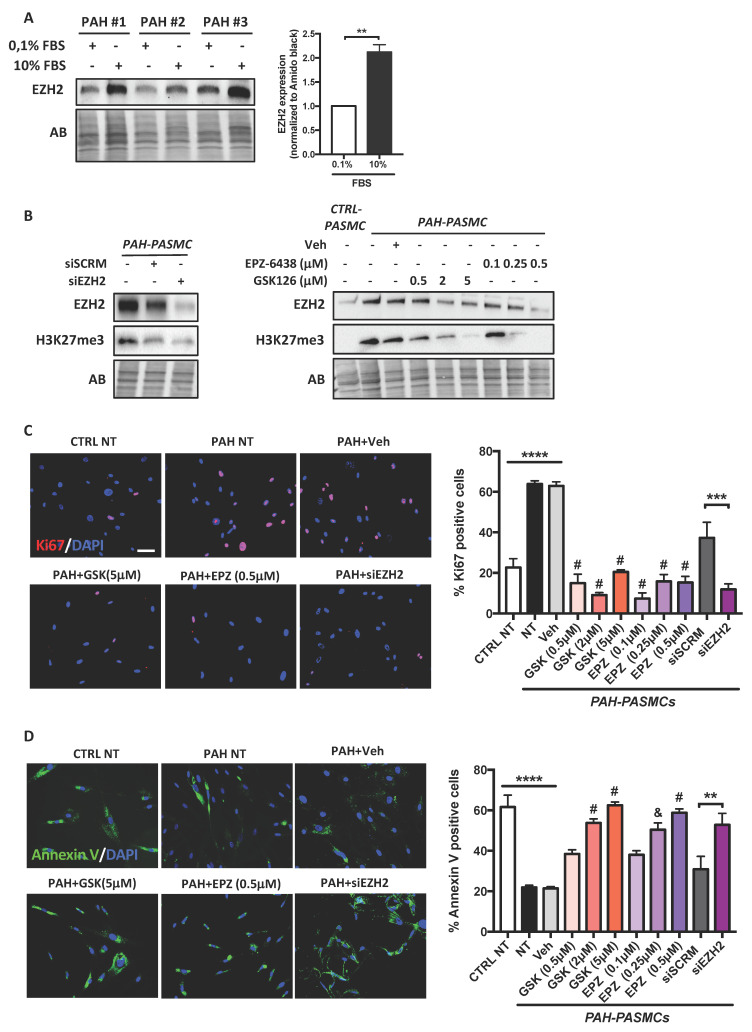
Effects of molecular and pharmacological inhibition of EZH2 on PAH-PASMCs proliferation and resistance to apoptosis. (**A**) Western blot and corresponding densitometric analysis of EZH2 in PAH-PASMCs that were serum-starved or not for 48 h; (**B**) Representative Western blots of EZH2 and H3K27me3 in PAH-PASMCs treated with either EZH2 small interference siRNA or nontargeting scrambled (siSCRM) or escalating concentrations of GSK126 and EPZ-6438 (two catalytic EZH2 inhibitors) for 48 h; (**C**) Proliferation (Ki67) was measured in control (*n* = 5) and PAH-PASMCs (*n* = 5) treated or not with siEZH2, GSK126, and EPZ-6438 or their respective controls for 48 h. Graph shows the percentage of cells with positive nuclear Ki67 staining. PAH-PASMCs are significantly more proliferative than control PASMCs. Pharmacological inhibition of EZH2 or its knockdown decreases PAH-PASMC proliferation; (**D**) Apoptosis (Annexin V) was measured in serum-starved control (*n* = 5) and PAH-PASMCs (*n* = 5) treated or not with siEZH2, GSK126, and EPZ-6438 or their respective controls for 48 h. Graph shows the percentage of cells with positive Annexin-V staining. PAH-PASMCs are more resistant to starvation-induced apoptosis than control cells. Pharmacological inhibition of EZH2 or its knockdown increases PAH-PASMC apoptosis. Scale bar = 50 μm. Data are presented as mean ± SEM; ** *p* < 0.01; *** *p* < 0.001; **** *p* < 0.0001; and ^&^ *p* < 0.001 compared with Veh and ^#^ *p* < 0.0001 compared with Veh.

To investigate whether upregulation of EZH2 contributes to the abnormal phenotype of PAH-PASMCs, we first measured EZH2 expression levels in response to serum starvation and serum stimulation. We found that EZH2 is increased in serum-fed proliferating cells compared to serum-deprived quiescent cells (Figure 2A), indicating that EZH2 abundance is intimately associated with the proliferative capacity of cells. We next exposed PAH-PASMCs to escalating doses of EPZ-6438 (Tazemetostat) and GSK-126 for 48 h; two selective S-adenosyl methionine competitive inhibitors of histone methyl transferase EZH2 currently being evaluated in clinical trials for the treatment of cancer [17,18]. As expected, treatment with EPZ-6438 or GSK-126 dose-dependently reduced overall H3K27me3 levels in PAH-PASMCs (Figure 2B). We found that both compounds elicited marked antiproliferative effects, as demonstrated by a sharp reduction in the percentage of cells positive for Ki67 or EdU (Figure 2C and Figure S2A). Moreover, using Annexin V labeling as a readout of early-stage apoptosis, EPZ-6438 and GSK-126 dose-dependently reverted the apoptosis-resistant phenotype of PAH-PASMCs (Figure 2D). These effects were accompanied with reduced levels of the proliferative and antiapoptotic markers minichromosome maintenance protein 2 (MCM2) and Survivin, respectively (Figure S2B). Because these pharmacological inhibitors might have off-target effects, we next examined the impact of EZH2 silencing using siRNA. Compared to scrambled siRNA transfected PAH-PASMCs, cells transfected with EZH2 siRNAs had significantly reduced EZH2 protein expression levels, indicating EZH2 knockdown efficiency (Figure 2B). As observed with pharmacological inhibitors, the hyperproliferation and resistance to apoptosis of PAH-PASMCs was diminished upon endogenous EZH2 depletion (Figure 2C,D and Figure S2). As a complementary approach, we sought to determine whether EZH2 gain-of-function is sufficient to enhance proliferation of control PASMCs and render them resistant to apoptosis. To this end, PASMCs isolated from control patients were infected with an adenovirus encoding human EZH2 for 48 h. Increased expression of EZH2 protein was observed in cells transfected with Ad-EZH2 compared with noninfected cells or cells infected with empty adenoviral vector (Ad-Null) (Figure S2C). As revealed by Ki67 labeling and EdU incorporation, upregulation of EZH2 in control cells significantly promoted their proliferation (Figure S2D). Moreover, overexpression of EZH2 markedly reduced serum starvation-induced apoptosis in control PASMCs (Figure S2D). Collectively, these data demonstrate that EZH2 plays a prominent role in the abnormal phenotype of PAH-PASMCs.

### 2.3. Impact of Molecular Inhibition of EZH2 on the PAH-PASMC Transcriptome

**Figure 3:**
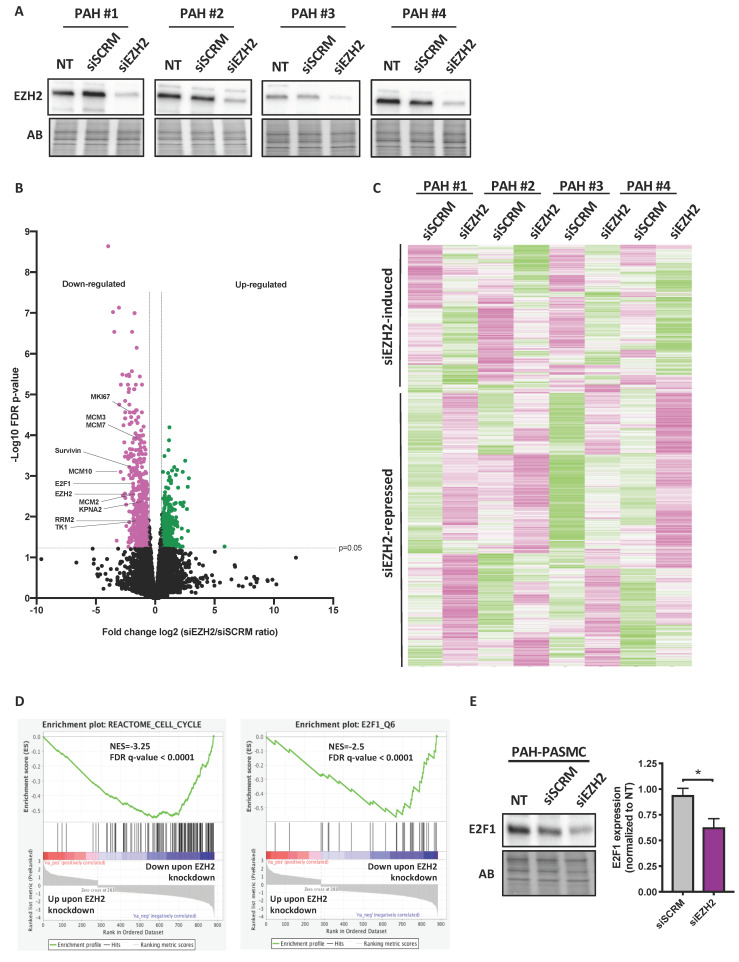
EZH2 knockdown in PAH-PASMCs and quantification of global transcript abundances by RNA sequencing. (**A**) Western blots of EZH2 in four different PAH-PASMC cell lines transfected or not with a siRNA targeting EZH2 (siEZH2) or a scrambled control siRNA (siSCRM). EZH2 siRNA markedly reduces EZH2 levels in the four PAH-PASMC cell lines; (**B**) Volcano plot comparing the transcriptome of siSCRM and siEZH2-treated PAH-PASMCs. Vertical lines indicate a fold change ± 1.5 and the horizontal line is indicative of a false-discovery-rate value threshold of 0.05. Pink (downregulated) and green (upregulated) points: transcripts that meet both criteria for significant change (i.e. FDR < 0.05 and fold change > 1.5) between control and EZH2-depleted cells; (**C**) Heatmap representation of all mRNAs whose expression levels were significantly impacted (FDR < 0.05) after silencing of EZH2 by a fold change > 1.5 or <1.5. The columns represent the results of four independent PAH-PASMC cell lines transfected with siSCRM and siEZH2. Rows show individual differentially expressed genes. The color key from pink to green represents transcript abundance from low to high. Selected genes are marked on the right side; (**D**) Selected gene set enrichment analysis (GSEA) charts showing enrichment for genes in the cell cycle process and E2F1 targets. The top portion of each panel represents the normalized enrichment score (NES) for each gene; the bottom portion of the plot shows the value of the ranking metric moving down the list of ranked genes; (**E**) Western blot showing expression levels of E2F1 in PAH-PASMCs subjected or not to EZH2 knockdown for 48 h. Quantitative densitometric analysis is shown. Protein expression was normalized to Amido black (AB). Data are presented as mean ± SEM; * *p* < 0.05.

To identify the downstream targets and signaling pathways governed by EZH2 and accounting for the abnormal phenotype of PAH-PASMCs, we examined global gene expression changes due to EZH2 knockdown by comparative RNA sequencing (RNA-Seq) analyses. These experiments were conducted in four different PAH-PASMCs, for which efficient EZH2 knockdown was previously examined by Western blot (Figure 3A). Using a fold-change cutoff value of 1.5 with FDR < 0.05, 884 differentially expressed genes (DEG) were identified (Figure 3B,C). Among them, 601 genes were significantly downregulated in EZH2-depleted cells compared to nontargeting siRNA controls. Based upon the RNA-Seq data, we ranked all genes by their fold change between EZH2 knockdown versus control cells and performed gene set enrichment analysis (GSEA) to identify biological pathways enriched among the most up- or down-regulated mRNAs. Analysis revealed that the downregulated genes in siEZH2-treated PAH-PASMCs were particularly enriched in the cell proliferation-related processes (Figure 3D). Interestingly, our data displayed significant enrichment of a gene set regulated in prostate cancer cell after siRNA-mediated depletion of EZH2 (Figure S3A) [19]. The identified up- and down-regulated DEGs were then separately subjected to gene ontology (GO) and Kyoto Encyclopedia of Genes and Genomes (KEGG) pathway enrichment analyses using the online database for annotation, visualization, and integrated discovery (DAVID). The results of the GO analysis revealed that the downregulated DEGs were mainly enriched in biological processes including “DNA replication initiation”, “mitotic cytokinesis”, and “cell division” (Figure S3B). Indeed, expression levels of multiple genes found to be upregulated and implicated in the pro-proliferative and apoptosis resistant phenotype of PAH-PASMCs were downregulated in siEZH2-treated cells. These include members of the minichromosome maintenance (MCM) family (MCM2 to MCM7), pituitary tumor-transforming gene 1 (PTTG1), ribonucleotide reductase regulatory subunit M2 (RRM2), thymidine kinase 1 (TK1), and Survivin [20,21]. In terms of molecular function, the downregulated DEGs were enriched in “protein binding”, “microtubule binding”, and “DNA helicase activity” (Figure S3B). Accordingly, KEGG pathway analysis of the downregulated genes demonstrated a significant representation of those involved in cell cycle and DNA replication (Figure S3B), supporting the GSEA analysis. On the other hand, there were no enriched categories of GO functional annotations for upregulated genes, and enrichment of biological pathways supplied by KEGG was limited to Pi3-Akt signaling and ECM-receptor interaction (Figure S3B).

Based on our data showing that most of the DEGs are decreased upon EZH2 knockdown and recent studies showing that EZH2 can cooperate with various transcription factors to stimulate gene transcription [14,22], we thus hypothesized that the downstream effects of EZH2, may be mediated, in part, by a transcription factor. We thus conducted GSEA analysis using the C3 transcription factor targets database. As shown in Figure 3D, rank-based analysis revealed a strong negative enrichment for the hallmark E2Fs targets signatures upon EZH2 knockdown, suggesting that EZH2 influences the transcriptome of the E2F pathway in PAH-PASMCs. In addition, consensus TF motif analysis unraveled by the Encyclopedia of DNA Elements (ENCODE) and ChIP enrichment analysis (ChEA) data sets determined using EnrichR revealed E2F members at top candidates with significant *p* values (Figure S4A). Consistently, E2F transcription factors are pivotal regulators of genes required for mitochondrial homeostasis, DNA repair, and cell-cycle progression [23,24]. The above findings pushed us to investigate whether members of the E2F family are significantly impacted by EZH2 knockdown. Among the DEGs, we found that only E2F1 was markedly decreased (logFC = −1,857; FDR = 0.001) (Figure 3B). Likewise, E2F1 protein levels were decreased in siEZH2-treated PASMCs (Figure 3D), suggesting involvement of E2F1 in the EZH2-mediated upregulation of gene expression. In support of this, we found that expression levels of well-established cell promoting factors and E2F1 targets genes, i.e., MCM2, RRM2, KPNA2, and PTTG1 [22], were diminished by EZH2 knockdown and further reduced in PAH-PASMCs simultaneously exposed to the pan-E2F inhibitor HLM006474 and siEZH2 (Figure S4B). Because EZH2 was documented to physically interacts and cooperate with E2F1 to stimulate the expression of a group of genes involved in cancer progression [15,22], we thus tested by co-immunoprecipitation whether such an interaction occurs in four different PAH-PASMC cell lines. Although EZH2 was found to interact with YY1 (a known binding partner of EZH2) as expected [25], no interaction between EZH2 and E2F1 was detected (Figure S4C), adding further evidence that physical interaction between EZH2 and E2F1 is not required for EZH2 to promote E2F1 signaling in PAH-PASMCs. Together with previous transcriptomic data, these findings point to a dominant role of increased EZH2 in stimulating pro-proliferative signaling pathways.

### 2.4. Analysis of siEZH2-Associated Proteome Changes by Quantitative Proteomics

**Figure 4:**
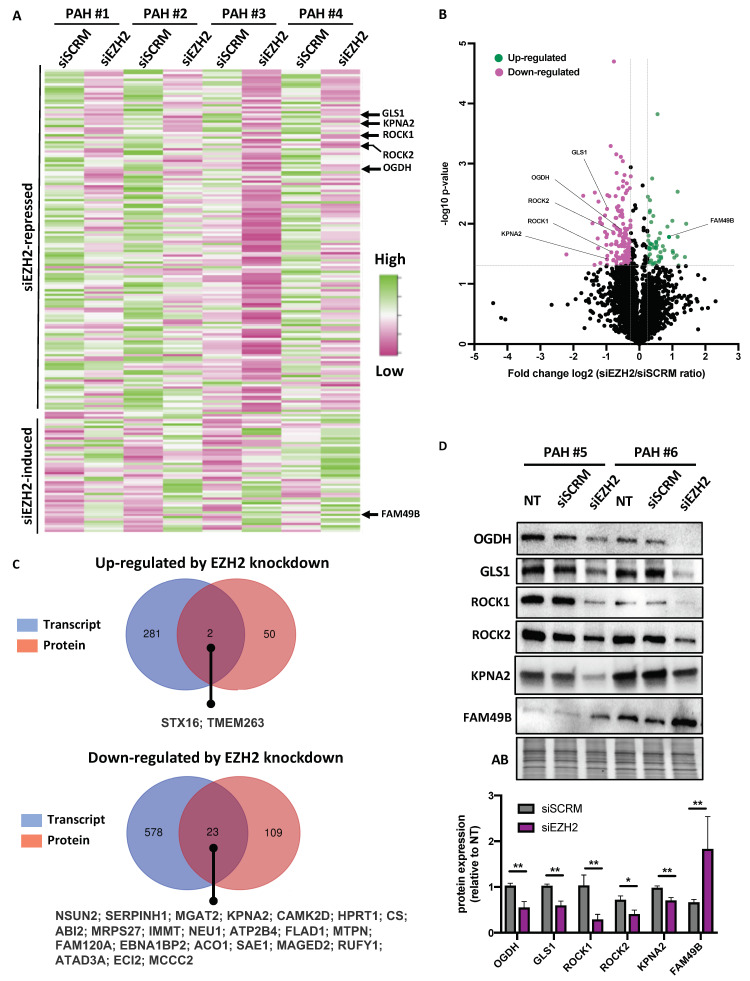
Proteomic analysis of siRNA-mediated silencing of EZH2 in PAH-PASMCs. (**A**) Heatmap representation of all proteins whose expression levels were significantly impacted (FDR < 0.05) after silencing of EZH2 by a fold change > 1.2 or <1.2. The columns represent the results of four independent PAH-PASMC cell lines transfected with siSCRM and siEZH2. Rows show individual differentially expressed proteins. The color key from pink to green represents protein abundance from low to high. Selected proteins are marked on the right side; (**B**) Volcano plot comparing the proteome of siSCRM and siEZH2-treated PAH-PASMCs. Vertical lines indicate a fold change ± 1.2, and the horizontal line is indicative of a paired t-test *p*-value threshold of 0.05. Pink (downregulated) and green (upregulated) points: transcripts that meet both criteria for significant change (i.e. paired t-test *p*-value < 0.05 and fold change > 1.5) between control and EZH2 depleted cells; (**C**) Venn diagrams showing the numbers of common and unique differentially expressed genes (blue) and differentially expressed proteins (red) found to be significantly up- and down-regulated upon EZH2 knockdown in PAH-PASMCs; (**D**) Validation of proteomic results by Western blot. Representative immunoblots and corresponding densitometric analysis of selected proteins (OGDH, GLS1, ROCK1, ROCK2, KPNA2, and FAM48B) in PAH-PASMCs (*n* = 6–10) subjected or not to EZH2 knockdown for 48 h. Protein expression was normalized to Amido black (AB). Data are presented as mean ± SEM; * *p* < 0.05 and ** *p* < 0.01.

To further dissect the EZH2 downstream signaling pathways in PAH-PASMCs, an LC-MS/MS proteomics approach was applied to appreciate global protein abundance changes in PAH-PASMCs treated or not with siEZH2. These experiments were conducted in the same four different PAH-PASMCs used for RNA-Seq analysis for which efficiency of EZH2 knockdown was previously confirmed (data not shown). Using a fold change > 1.2 and a *p* value < 0.05, we identified 184 differentially expressed proteins (DEPs) upon EZH2 knockdown (Figure 4A,B). Of these 184 DEPs, 132 were downregulated, and 52 were upregulated upon EZH2 depletion (Figure 4A,B). Not surprisingly, only a small number of genes was found to be dysregulated at both the transcription and translation levels with all these genes exhibiting the same direction of change at the two levels (Figure 4C). To confirm the robustness and sensitivity of the expression changes observed in our proteomic data sets, expression levels of six DEPs (ROCK1, ROCK2, OGDH, GLS, KPNA2, and FAM49B), already known to be implicated in the pro-proliferative and apoptosis-resistant phenotype of PAH-PASMC or cancer cells [6,20,26,27], were measured by Western blotting in six different PAH-PASMC lines (including the four used in our unbiased proteomic approach). Consistent with the results of LC-MS/MS proteomics, ROCK1, ROCK2, KPNA2, OGDH, and GLS were significantly downregulated in siEZH2-treated PAH-PASMCs, whereas FAM49B was augmented (Figure 4D). Interestingly, no significant modulation of these factors was seen upon exposure to EPZ-6438, indicating that EZH2 regulates these genes in a PRC2-independent manner and supporting the notion that EZH2 mediates dual transcription programs in PAH-PASMCs (Figure S5A,B). To get a better insight into the biological significance of these DEPs, both up- and down-regulated proteins were subject to GO enrichment analysis using the DAVID database. We found that downregulated proteins in siEZH2-treated cells were significantly enriched in pathways involved in tricarboxylic acid (TCA) cycle and mitochondrial translation (Figure S5C). In the cell component ontology, most of the categories that satisfy the cutoff criteria of Benjamini–Hochberg adjusted *p* < 0.01 were associated with the mitochondria (Figure S5C). By contrast, no enriched categories of GO functional annotation nor KEGG pathways were found for upregulated proteins.

**Figure 5:**
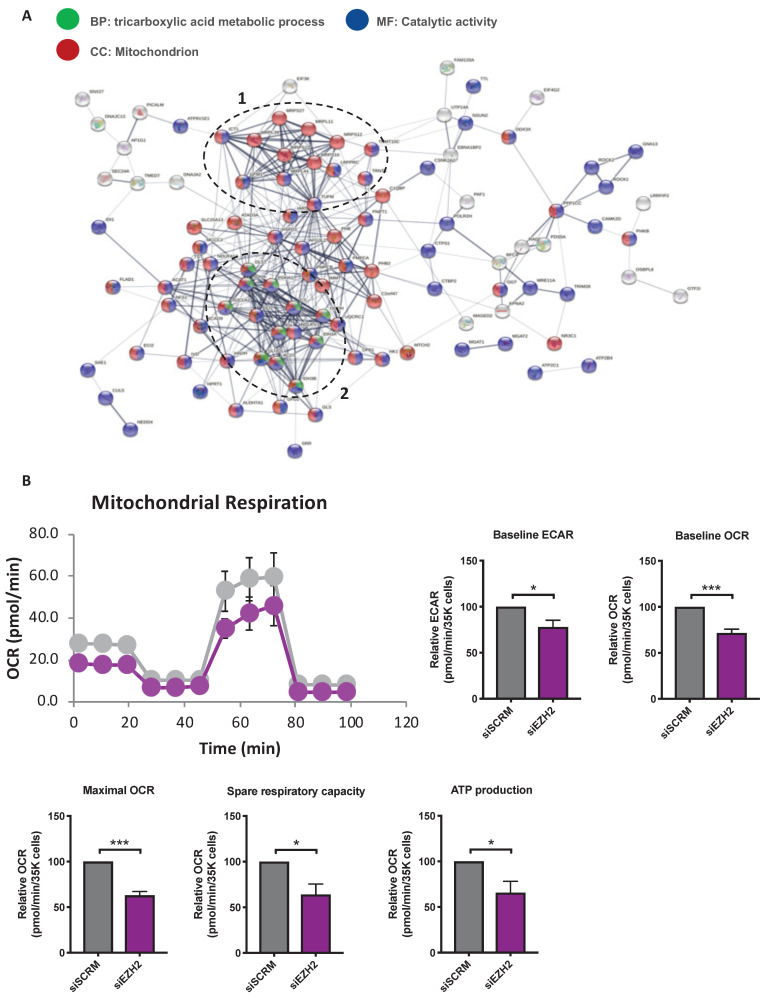
Impact of EZH2 suppression on PAH-PASMC bioenergetics. (**A**). Network analysis via STRING of the proteins found to be downregulated following EZH2 knockdown, revealing the presence of two major clusters of proteins associated with (1) mitochondrial gene expression and (2) glutamine-dependent biosynthetic pathway and tricarboxylic acid (TCA) cycle. Proteins belonging to the top enriched biological process (i.e. TCA cycle metabolism), molecular function (i.e. catalytic activity), and cellular component (i.e. mitochondrion) are colored in green, blue, and red, respectively. Proteins (nodes) with no predicted interactions were excluded. Thickness of the connecting lines correspond to the confidence level. Labels report protein names; (**B**) Assessment of mitochondrial function by the Seahorse XF96 analyzer in PAH-PASMCs exposed to siEZH2 or siSCRM for 48 h. The basal extracellular acidification rate (ECAR), basal oxygen consumption rate (OCR), maximal OCR, spare respiratory capacity, and ATP production were quantified following indicated injection of the specific stressors oligomycin (1 μM), carbonyl cyanide-4-(trifluoromethoxy) phenylhydrazone (FCCP, 5 μM), and rotenone (1 μM). Seahorse data were normalized to the total cell numbers plated in each well. Experiments were performed in triplicate in three different PAH-PASMC cell lines. Data are presented as mean ± SEM; * *p* < 0.05 and *** *p* < 0.001.

To further investigate the protein–protein interaction network among the downregulated proteins in both four biological replicates, a comprehensive interaction network of the 134 DEPs was performed through the String website. Following the elimination of nodes with no predicted interactions, a network of DEGs was constructed (Figure 5A). Two highly connected clusters of protein nodes emerged. Cluster 1 was composed of proteins required for mitochondrial gene expression, including factors involved in mRNA maturation and stability (TRMT10C and LRPPRC), translational elongation factors (GFM1 and TUFM), and mitoribosomal proteins of the small (MRPS) and large (MRPL) subunits (MRPS12, MRPS16, MRPS27, MRPL11, MRPL44, and MRPL58 (also called ICT1)). The second cluster comprises factors involved in glutamine-dependent biosynthetic pathway and TCA cycle, including GLS, GLUD1, CS, SDHA, OGDH, IDH3A/B, and SUCLA2 (Figure 5A). Data derived from proteomic analysis prompted us to examine functional assessment of the PAH-PASMC mitochondria upon EZH2 knockdown using the Seahorse assay. Suppression of EZH2 significantly diminished the glycolytic capacity of PAH-PASMCs after 48 h (Figure 5B). Moreover, basal oxygen consumption rate (OCR), a surrogate marker of OXPHOS activity, was significantly impaired (Figure 5B). Subsequent measurements with application of inhibitors and uncouplers demonstrated that siRNA-mediated silencing of EZH2 significantly reduced ATP production, maximal respiration and spare respiratory capacity in comparison to siSCRM-treated cells (Figure 5B). Altogether, these results suggest that siEZH2-induced altered mitochondrial function and associated bioenergetics defects likely contribute to reduced PAH-PASMC proliferation and survival.

## 3. Discussion

Originally described as the catalytic subunit of the PRC2, EZH2 mediates H3K27 trimethylation and gene silencing. Over the last decade, numerous studies have demonstrated that increased expression of EZH2 is a common denominator of multiple cancers promoting uncontrolled cell proliferation, at least in part, through transcriptional repression of critical target genes [9,14]. More recently, noncanonical oncogenic functions of EZH2 have been documented. Indeed, a growing number of studies indicate that EZH2 can also methylate nonhistone proteins to regulate their activity or half-life [11,28] and serves as a coactivator of several pro-oncogenic transcription factors such as androgen receptor [13], NF-kB subunits [29], SWI/SNF [30], and E2F members [15,22,31] to name a few. The importance of the oncogenic role of EZH2 independent of its methyltransferase activity was further underscored in natural killer/T-cell lymphoma in which ectopic expression of an EZH2 mutant form lacking methyltransferase activity was able to confer a growth advantage as well as to rescue growth inhibition as a result of endogenous EZH2 depletion [32]. Furthermore, studies have revealed that the canonical and noncanonical functions of EZH2 are not mutually exclusive but coexist in the same cell [30], pinpointing the cell-context-dependent and multifaceted effects of EZH2 on tumor progression. Consistent with the fact that PAH-PASMCs express many protumorigenic factors, we found that increased EZH2 expression is a shared feature of human PAH and animal models. More importantly, we demonstrated that blockade of EZH2 methyltransferase activity using EPZ-6438 and GSK126 markedly impedes the pro-proliferative and apoptosis-resistant state of PAH-PASMCs; the findings were recapitulated by EZH2 knockdown using siRNAs. To rule out possible off-target effects of EZH2 pharmacological inhibitors and gain some meaningful insights into the downstream signaling pathways governed by EZH2, high-throughput RNA sequencing, and quantitative proteomics were performed in four different PAH-PASMCs cell lines subjected to EZH2 silencing using siRNA. Surprisingly, we found that a large set (nearly 70%) of transcripts and proteins differentially expressed upon EZH2 silencing were downregulated. This result likely indicates that effects of EZH2 on gene expression are, in a large part, mediated by noncanonical functions, as inhibition of its catalytic function is expected to relieve transcriptional repression and thus promote gene expression. Furthermore, we showed that selected proteins significantly down- or up-regulated upon EZH2 knockdown were not impacted by EPZ-6438. In support of this, by comparing the transcriptome of human prostate adenocarcinoma cells treated with siEZH2 or EPZ-6438, Kim and colleagues found that the majority of siEZH2 downregulated genes were unaffected by EPZ treatment [14].

Although it is generally assumed that a high concordance exists across transcriptome and proteome data sets, numerous studies have revealed that transcriptomic-proteomic modestly overlap challenging this dogma [33,34]. In the present study, a low level of concordance was found between changes in mRNA expression and protein abundance levels. Indeed, global proteomic analysis identified impaired mitochondrial translation and associated defects in oxidative phosphorylation as the main biological processes altered in EZH2-depleted PAH-PASMCs, whereas downregulated genes in siEZH2-treated cells were mainly enriched in functions linked to cell-cycle progression and cell division, indicating that changes in mRNA expression provide only limited insight into the downstream mechanisms regulated by EZH2. Several factors may explain this low correlation, including intermediate transcriptional and post-transcriptional regulatory mechanisms, experimental timing, and limited sensitivity of mass spectrometry. In this regard, Survivin and E2F1, found to be significantly downregulated by RNA-Seq, were significantly decreased by Western blot but not by LC-MS/MS proteomics. Importantly, we found that numerous transcripts of genes already known to be implicated in the pro-proliferative and apoptosis-resistant phenotype of PAH-PASMCs, such as Survivin [21], ADAMTS8 [35], PBK [20], TK1 [36], and E2F1 [37], were significantly repressed under EZH2 inhibition, which is consistent with the hypothesis that EZH2 plays a critical role in the abnormal phenotype of disease cells. Furthermore, several factors implicated in the protection of DNA damage or in DNA repair itself were diminished. These include NUDT1, RAD51, LIG1, CLSPN, and XRCC3. In a prior study, depletion of EZH2 was documented to decrease the efficiency of double-strand-break repair and increases sensitivity of cells to DNA-damaging agents [38]. As we previously demonstrated that PAH-PASMCs have developed multiple complementary mechanisms dedicated to preserve genome integrity [39,40,41], siEZH2-induced inhibition of these factors may collectively interfere with the PAH-PASMC DNA repair capacity, thus impairing cell proliferation and survival. Interestingly, we also observed a diminution in the abundance of a large array of proteins implicated in mitochondrial translation and TCA in siEZH2-treated PAH-PASMCs and accordingly reduced OXPHOS capacity. This confirms previous data showing that EZH2 silencing in glioblastoma cells reduces oxygen consumption rates [42].

As observed in cancer cells, PAH-PASMCs are characterized by a metabolic reprogramming towards aerobic glycolysis (also called Warburg effect) allowing the generating of a large amount of ATP and biosynthetic precursors necessary to sustain their growth [5]. Despite enhanced glycolysis, oxidative phosphorylation remains essential for disease cell survival [41,43], making oxidative phosphorylation an emerging target in hyperproliferating disease. Proteins encoded by the mitochondrial DNA are essential for oxidative phosphorylation and thus, energy production. Therefore, it can be assumed that defective expression of nuclear-encoded components of the mitochondrial translation machinery largely contributes to the pro-apoptotic and antiproliferative effects of EZH2 knockdown. In agreement with this, emetine, a cytoplasmic translation inhibitor, was recently documented to ameliorate pulmonary hypertension in two animal models by reducing the expression of Rho-kinases and surviving in PAH-PASMCs [44]. Finally, upregulation of the mitochondrial-localized protein FAM49B [27] was seen in EZH2-inhibited PAH-PASMCs. Although the molecular functions of the latter remain largely unknown, FAM49B was reported to act as a tumor suppressor by regulating mitochondrial integrity and metabolism [27].

Taken together, it is tempting to speculate that inhibition of EZH2 may exert beneficial effects in improving pulmonary vascular remodeling in PAH. Nevertheless, EZH2 loss-of-function targeted to RV cardiomyocytes was shown to induce cardiac hypertrophy and fibrosis [45]. In addition, we recently reported a dramatic downregulation of EZH2 in decompensated RV from PAH patients and animal models as well as an increased expression of EZH2 in animals presenting improved cardiac function secondary to therapeutic intervention [16]. These findings strongly suggest that interfering with EZH2 function may induce detrimental effects in the vulnerable PAH right ventricle and that specific delivery of EZH2 inhibitor to target cells should be envisioned. Further research is needed to better understand the complexity of EZH2 and the relative contribution of its PRC2-dependent and independent roles in the abnormal phenotype of PAH-PASMCs, a prerequisite for developing the most relevant therapeutic approach.

In conclusion, we provide evidence that increased expression of EZH2 contributes to the hyperproliferative and apoptosis-resistant phenotype of PAH-PASMCs through both canonical and noncanonical mechanisms. By combining transcriptomic and proteomic analysis in siEZH2-depleted PAH-PASMCs, we identified disease-relevant functions of EZH2 in maintaining the bioenergetic machinery of oxidative phosphorylation and stimulating expression of genes associated with cell-cycle progression and survival; providing a useful resource that can be exploited to identify new actionable targets to improve pulmonary vascular remodeling in the setting of PAH.

## 4. Materials and Methods

### 4.1. Human Lung Samples and Animal Models

Experimental procedures using human tissues or cells conformed to the principles outlined in the Declaration of Helsinki. Written informed consent was obtained for all subjects and the study was approved by the IUCPQ-UL ethics committee (CER #20773). Clinical and hemodynamic characteristics of patients are shown in Table S1. Animal experiments were approved by the Institut Universitaire de Cardiologie et de Pneumologie de Québec—Université Laval Biosafety and Ethics Committees (#2019-018). To induce pulmonary hypertension, adult Sprague Dawley rats (Charles River laboratories, Laval, QC, Canada) were injected with monocrotaline (MCT, 60 mg/kg s.c.) or injected with SU5416 (20 mg/kg s.c.) before exposition to normobaric hypoxia for 3 weeks, as previously described [20]. Experiments were terminated 4 and 5 weeks after MCT or SU5416 injection, respectively. The animals were sacrificed, and the lungs were collected for analysis.

### 4.2. Cell Culture and Treatments

PAH-PASMCs (*n* = 11 cell lines) were isolated from small PAs (<1000 µm diameter) from PAH patients. Control PASMCs (*n* = 10 cells lines) were either purchased from Cell Application (San Diego, CA, USA) or isolated from non-PAH patients (Table S2). PASMCs were grown in high-glucose DMEM supplemented with 10% fetal bovine serum (FBS) and containing 1% penicillin/streptomycin. The purity of the PASMCs in the primary cultures was confirmed by staining for alpha smooth muscle actin (αSMA) using immunofluorescence technique. Only cells between passages 3 to 9 were used for experiments. EPZ-6438 and GSK-126 were purchased from SelleckChem (Burlington, ON, Canada), dissolved in dimethyl sulfoxide (DMSO), and then added to the culture medium at the indicated concentrations immediately before use. The E2F inhibitor, HLM006474, was purchased from MedChemExpress (Monmouth Junction, NJ, USA) and dissolved in DMSO. Human EZH2 and null adenoviruses were provided by Vector Biolabs (Burlington, ON, Canada). Infection was performed at a multiplicity of infection of 100 plaque-forming units for 48 h before harvesting and analysis. PAH-PASMCs were transfected with silencer RNA targeting EZH2 mRNA (Cat#S102665166, Qiagen, final concentration of 10 nM) using lipofectamine RNAiMAX reagent (Thermo Fischer Scientific, Saint-Laurent, QC, Canada) according to the manufacturer’s protocol. siRNA with scramble sequence (siSCRM) was used as negative control siRNA. Cells were cultured for 48 h before collecting material for transcriptomic, proteomic, and immunofluorescence analysis.

To assess cell proliferation and resistance to apoptosis, control and PAH-PASMCs were cultured for 48 h in 10% fetal bovine serum (FBS, a condition that is known to promote proliferation) or 0.1% FBS (a starvation condition that promotes apoptosis) [46,47]. Cell proliferation was determined with either Ki67 labeling or 5-Ethynyl-2’-deoxyuridine (EdU) incorporation assay (Click-iT EdU assay kit, Thermo Fischer Scientific, Saint-Laurent, QC, Canada) according to the manufacturer’s instructions. Briefly, EdU was added during the last 2 h. After incubation, EdU-positive DNA duplicating cells were fixed with 3.7% formaldehyde diluted in PBS1X for 15 min at room temperature, washed with PBS1X, and then permeabilized during 20 min in 0.5% Triton X-100 in PBS. After washing in 3% BSA in PBS1X, cells were stained with the Click-iT reaction mix for 30 mins and counterstained with DAPI. Apoptosis was evaluated by Annexin V assay, as previously described [41]. The Ki67/EdU proliferative and Annexin V apoptotic index were calculated by counting the number of positive-staining cells divided by the total number of DAPI-positive cells multiplied by 100. For each cell line, experiments were performed in triplicate, and at least 500 cells per condition were counted.

### 4.3. Real-Time Quantitative PCR

Total RNA was isolated from human PASMCs using Trizol reagent (Invitrogen) according to the manufacturer’s instructions. The quality and concentration of total RNA were then determined with a spectrophotometer (NanoDrop 2000, Thermo Fischer Scientific, Saint-Laurent, QC, Canada). RNA integrity was confirmed by electrophoresis on a denaturing agarose gel. Complementary DNA (cDNA) was synthesized using the qScript Flex cDNA Synthesis Kit (Quanta bio, Beverly, MA, USA). Real-time PCR was carried out in a QuantStudio 7 Flex real-time PCR system (Thermo Fischer Scientific, Saint-Laurent, QC, Canada) using SsoAdvanced Universal SYBR Green Supermix (Bio-Rad Laboratories, Mississauga, ON, Canada). Each sample was analyzed in triplicate. Gene expression levels were normalized to the housekeeping gene 18S. Relative expression levels were calculated using the ΔΔCt method. Primer sequences are listed in Table S3.

### 4.4. RNA Sequencing

RNA-sequencing analysis was carried out on total RNA extracted from PAH-PASMCs with or without siRNA-mediated silencing of EZH2. Qualitative and quantitative analysis of RNA was performed using NanoDrop2000 spectrophotometer (Thermo Scientific, Saint-Laurent, QC, Canada). Library preparation and paired-end RNAseq were performed by Cofactor genomics (St. Louis, MO, USA) using Illumina HiSeq, on the PASMC samples at a read length of 150 bp and an average read depth of 50 million read pairs. Adapters were removed by the sequencing provider The raw fastq files were examined for sequencing quality and adapter contamination using FastQC (http://www.bioinformatics.babraham.ac.uk/projects/fastqc, accessed on 3 June 2020), after which alignment to the human transcriptome was performed using STAR [48]. After the alignment was read, quantification of the reads at the transcript level were performed using RSEM [49], and gene level quantification was obtained by the summarizeToGene function in the tximport R package [50]. Genes/transcripts that had low or no counts were filtered out using the filterByExpr function implemented in the edgeR Bioconductor package [51,52]. Following this sample specific normalization factors were estimated using the calcNormFactors function, while accounting for the treatment and control design of the study. Differential expression analysis was performed using the voom-limma framework [53], and an FDR-corrected *p*-value cutoff of 0.05 was used to determine the significantly differentially expressed genes/transcripts. High-throughput sequencing data used in this study have been deposited in NCBI’s gene expression omnibus [54] and are accessible through GEO Series accession number GSE166996.

### 4.5. Proteomics

#### 4.5.1. Protein in-Gel Digestion

Cells were lysed and protein concentration was determined using the Bradford assay (Bio-Rad). Protein samples (10 µg) were reduced in Laemmli buffer for 5 min at 95 °C and then loaded on a homemade one-dimensional SDS-PAGE gel (8% separating gel overlaid with a 5% stacking gel). Electrophoresis was stopped as soon as the protein sample entered the separating gel. The gel was fixed, stained with Coomassie Blue R250, and a single band containing the whole sample was excised from the gel, placed in 96-well plate, and then washed with water. Tryptic digestion was performed on a MassPrep liquid handling robot (Waters, Milford, MA, USA) according to the manufacturer’s specifications and to the protocol of Shevchenko et al [55] with the modifications suggested by Havlis et al [56]. Briefly, proteins were reduced with 10 mM DTT and alkylated with 55 mM iodoacetamide. Trypsin digestion was performed using 126 nM of modified porcine trypsin (sequencing grade, Promega, Madison, WI, USA) at 37 °C overnight. Digestion products were extracted using 1% formic acid, 2% acetonitrile followed by 1% formic acid, and 50% acetonitrile. The recovered extracts were pooled, vacuum centrifuge dried, and then resuspended at 0.4 µg/µL with 2% acetonitrile, 0.05% trifluoroacetic acid.

#### 4.5.2. NanoLC/MSMS Analysis

For each sample, 5 µL of resuspended peptide digestion (equivalent to 2µg peptides) was injected and separated by online reversed-phase (RP) nanoscale capillary liquid chromatography (nanoLC) and analyzed by electrospray mass spectrometry (ESI MS/MS). The experiments were performed with a Dionex UltiMate 3000 nanoRSLC chromatography system (Thermo Fisher Scientific/Dionex Softron GmbH, Germering, Germany) connected to an Orbitrap Fusion Tribrid ETD mass spectrometer (Thermo Fisher Scientific, San Jose, CA, USA) equipped with a nanoelectrospray ion source. Peptides were trapped at 20 μL/min in loading solvent (2% acetonitrile, 0.05% TFA) on a 5 mm length 300 μm I.D., 5 µm particles Acclaim™ PepMap™ 100 precolumn cartridge (Thermo Fisher Scientific/Dionex Softron GmbH, Germering, Germany) during 5 min. Then, the precolumn was switched online with 500 mm length, 75 μm I.D., 3 µm particles, Acclaim™ PepMap™ 100 C18 analytical column (Thermo Fisher Scientific/Dionex Softron GmbH, Germering, Germany), and the peptides were eluted with a linear gradient from 5–40% solvent B (A: 0,1% formic acid, B: 80% acetonitrile, 0.1% formic acid) in 270 min, at 300 nL/min. Mass spectra were acquired using a data dependent acquisition mode using Thermo XCalibur software version 3.0.63. Full scan mass spectra (350 to 1800 *m*/*z*) were acquired in the orbitrap using an AGC target of 4e5, a maximum injection time of 50 ms, and a resolution of 120,000. Internal calibration using lock mass on the *m*/*z* 445.12003 siloxane ion was used. Each MS scan was followed by acquisition of fragmentation MSMS spectra of the most intense ions for a total cycle time of 3 s (top speed mode). The selected ions were isolated using the quadrupole analyzer in a window of 1.6 *m*/*z* and fragmented by higher-energy collision-induced dissociation (HCD) with 35% of collision energy. The resulting fragments were detected by the linear ion trap in rapid scan rate with an AGC target of 1e4 and a maximum injection time of 50 ms. Dynamic exclusion of previously fragmented peptides was set for a period of 20 s and a tolerance of 10 ppm.

#### 4.5.3. Database Searching and Label Free Quantification

Spectra were searched against a human proteins database (Uniprot Complete Proteome—taxonomy *Homo sapiens*—29 March 2017) using the Andromeda module of MaxQuant software v. 1.5.5.1 [57]. Trypsin/P enzyme parameter was selected with two possible missed cleavages. Carbamidomethylation of cysteins was set as fixed modification, methionine oxidation, and acetylation of protein N-terminus as variable modifications. Mass search tolerance were 5 ppm and 0.6Da for MS and MS/MS, respectively. For protein validation, a maximum false discovery rate of 1% at peptide and protein level was used based on a target/decoy search. MaxQuant was also used for label-free quantification. The “match between runs” option was used with 20 min as alignment time window and 3 min as match time window values. Only unique and razor peptides were used for quantification. All other parameters were set at default values.

#### 4.5.4. Data Treatment and Statistical Analysis Related to Proteomics

The peptides.txt file generated by MaxQuant was used in R software v 3.4. The intensity values of each peptide in each sample were normalized using the median of all intensity values in each sample (normalization by column). Only peptides having at least two values in one of the two conditions to compare were considered as quantified. Other missing values were imputed using a noise value calculated as the first centile of all intensity values per sample. Quantifiable peptides were then aggregated into proteins using the leading razor protein accession number given by MaxQuant, and the intensity values of their corresponding peptides were summed for each sample. Only proteins with at least two quantified peptides were kept for further analysis. For each protein, a ratio between the two conditions to compare was calculated using the average of protein intensities in all samples of the same group. A paired Student’s t-test was finally performed in R software to determine the probability of variation (*p*-value) of each protein between two groups of samples while taking into account the original cell line of each sample. Proteins with a *p*-value < 0.05 and fold change > 1.2 were considered as significantly variant between the two conditions.

### 4.6. Bioinformatic Analysis

Pathway enrichment analysis of differentially expressed genes and proteins were performed were performed using a combination of pathway enrichment methods, including gene set enrichment analysis (GSEA) [58], and DAVID 6.7 Bioinformatics resource tools [59]. Significantly enriched GO terms and pathways were selected based on Benjamini *p*-value < 0.01. The protein–protein interaction (PPI) analysis of DEPs was performed with the String website (http://string-db.org, accessed on 3 June 2020). An interaction score > 0.4 (medium confidence score) was considered significant and the PPI was visualized.

### 4.7. Western Blotting and Immunoprecipitation

For Western blotting, proteins were extracted from tissues or cell pellets using a 2% Chaps lysis buffer supplemented with a protease inhibitor cocktail (Roche). Lysates were centrifuged at 16,000× *g* for 20 min at 4 °C, and the supernatant was collected as total protein. Protein concentrations were determined by the Bradford method. Equal amounts of proteins were separated by electrophoresis in SDS-polyacrylamide gels and transferred to polyvinylidene fluoride membranes. Membranes were subsequently blocked with either 5% nonfat dry milk or 5% goat serum in TBS-T buffer and incubated with primary antibodies (Table S4) in 3% BSA overnight at 4 °C. After being rinsed three times with TBS-T buffer, membranes were incubated with appropriate horseradish peroxidase (HRP)-conjugated secondary antibody for 2 h at RT in 5% nonfat milk or in 5% goat serum in TBS-T buffer. Antibodies were revealed using ECL reagents (Perkin–Elmer) and labelled proteins were detected with the imaging Chemidoc MP system (Bio-Rad Laboratories). Protein expression was quantified using the Image lab software (Bio-Rad Laboratories) and normalized to Amido black as previously described [41].

For the co-immunoprecipitation, cell proteins were extracted with RIPA buffer (10 mM Tris-HCL pH7.6, 150 mM NaCl, 1% NP40, 0.5% sodium deoxycholate, and 0.1% sodium dodecyl sulfate) containing protease inhibitor cocktail (Sigma-Aldrich). Surebeads magnetic beads (Bio-Rad Laboratories) were preincubated with an anti-EZH2 antibody or anti-IgG isotype control antibody for 4 h on a low-speed rotating shaker at room temperature. Then, protein extracts were mixed with anti-EZH2 antibodies bound to magnetic beads and incubated overnight at 4 °C on rotating shaker. The beads were magnetized using SureBeads magnetic rack, and the supernatant was discarded. Then, elution buffer was used to collect purified target protein for western blot analysis.

### 4.8. Immunofluorescence Studies

Paraffin-embedded lungs were serially sectioned at 5 µm. Lung sections were dewaxed and rehydrated in graded ethanol solutions. Once rehydrated, slides were subjected to antigen retrieval in citrate buffer (0.01 M, pH 6.0) in a microwaveable pressure cooker for 20 min. Sections were blocked with 5% goat serum for 2 h and then incubated with indicated primary antibodies in a humidified chamber overnight at 4 °C. After washes, sections were further incubated for 1 h at room temperature with appropriate fluorescent-dye conjugated secondary antibodies. The Cyanine 3 Tyramide Signal Amplification Kit (PerkinElmer, Woodbridge, ON, Canada) was used for EZH2 detection. Sections were mounted onto coverslips using DAPI (4′,6-diamidino-2-phenylindol) Fluoromount G mounting medium. Sections were examined by microscopy using an Axio Observer microscope (Zeiss), and images were acquired using Zen system (Zeiss).

### 4.9. In Vitro Metabolism (Seahorse XF24) Assays

Mitochondrial bioenergetics/function was assessed using the Seahorse XF24 Analyzer (Agilent Technologies, Saint-Laurent, QC, Canada), as previously published [41]. Twenty-four hours after transfection, PAH-PASMCs were seeded in Seahorse 24-well tissue culture plates at a density of 3.5 × 10^4^ cells/well and allowed to adhere for 24 h. Prior to the assay, cell confluence was confirmed the media was changed to unbuffered DMEM containing pyruvate and glutamine, and the cells were equilibrated for 1 h at 37 °C in a non-CO_2_ incubator. After measuring basal OCR and ECAR, the mitochondrial stress was carried out, and OCR was determined after sequential injections with oligomycin (1 μM), carbonyl cyanide p-trifluoromethoxyphenylhydrazone (FCCP, 5 μM), and rotenone (1 μM) according to the manufacturer’s protocol. OCR rates were automatically calculated and recorded by the Seahorse XF24 software. The maximum respiration capacity was calculated by using the OCR measurement after the addition of FCCP. The maximum glycolytic capacity was calculated by the ECAR measurement after oligomycin addition. Rates of OCR and ECAR were normalized to cell number per well.

### 4.10. Statistical Analysis

All analyses were performed using GraphPad Prism 6.0 (GraphPad, San Diego, CA, USA). The unpaired Student t-test and one-way analysis of variance (ANOVA) test were used for comparisons between two and two or more normally distributed groups, respectively. The Mann–Whitney and Kruskal–Wallis nonparametric tests were used to compare two or more non-normally distributed groups. A significance level inferior to 5% (*p* < 0.05) was considered statistically significant.

## Data Availability

The data presented in this study are available in this article and in the supplementary material.

## Supplementary Materials

The following are available online at https://www.mdpi.com/1422-0067/22/6/2957/s1.
