# Supplementary material for: Implication of EZH2 in the Pro-Proliferative and Apoptosis-Resistant Phenotype of Pulmonary Artery Smooth Muscle Cells in PAH: A Transcriptomic and Proteomic Approach
Source: Int J Mol Sci. 2021 Mar 15;22(6):2957. doi: 10.3390/ijms22062957 (PMC7999120; doi:10.3390/ijms22062957)
Supplement: Supplementary file 1 [file ijms-22-02957-s001.pdf]

Supplemental Data

Implication of EZH2 in the hyper-proliferative and apoptosis-resistant phenotype of pulmonary artery smooth muscle cells in PAH: a transcriptomic and proteomic approach.

Karima Habbout[1], Junichi Omura[1], Charifa Awada[1], Alice Bourgeois[1], Yann Grobs[1], Vinod Krishna[2], Sandra Breuils-Bonnet[1], Eve Tremblay[1], Ghada Mkannez[1], Sandra Martineau[1], Valérie Nadeau[1], Florence Roux-Dalvai[3], Mark Orcholski[1], Jey Jeyaseelan[2], David Gutstein[2], François Potus[1], Steeve Provencher[1,4], Sébastien Bonnet[1,4], Roxane Paulin[1,4] and Olivier Boucherat[1,4,*]

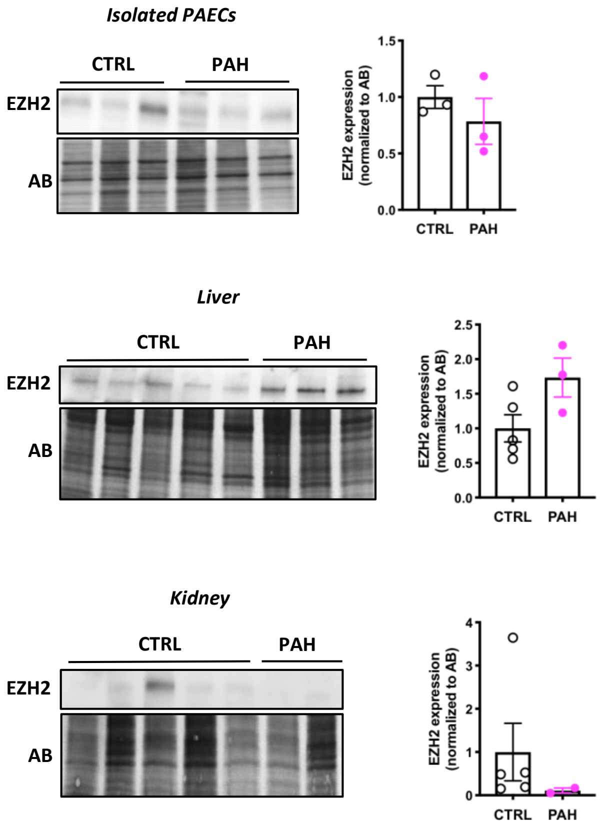

Figure S1. Expression of EZH2 in PA endothelial cells and tissues from control and PAH patients. Western blots and corresponding densitometric analyses of EZH2 in isolated pulmonary artery endothelial cells (PAECs), liver and kidney biopsies from control and PAH patients. Protein expression was normalized to Amido black (AB). Data are presented as mean±SEM.

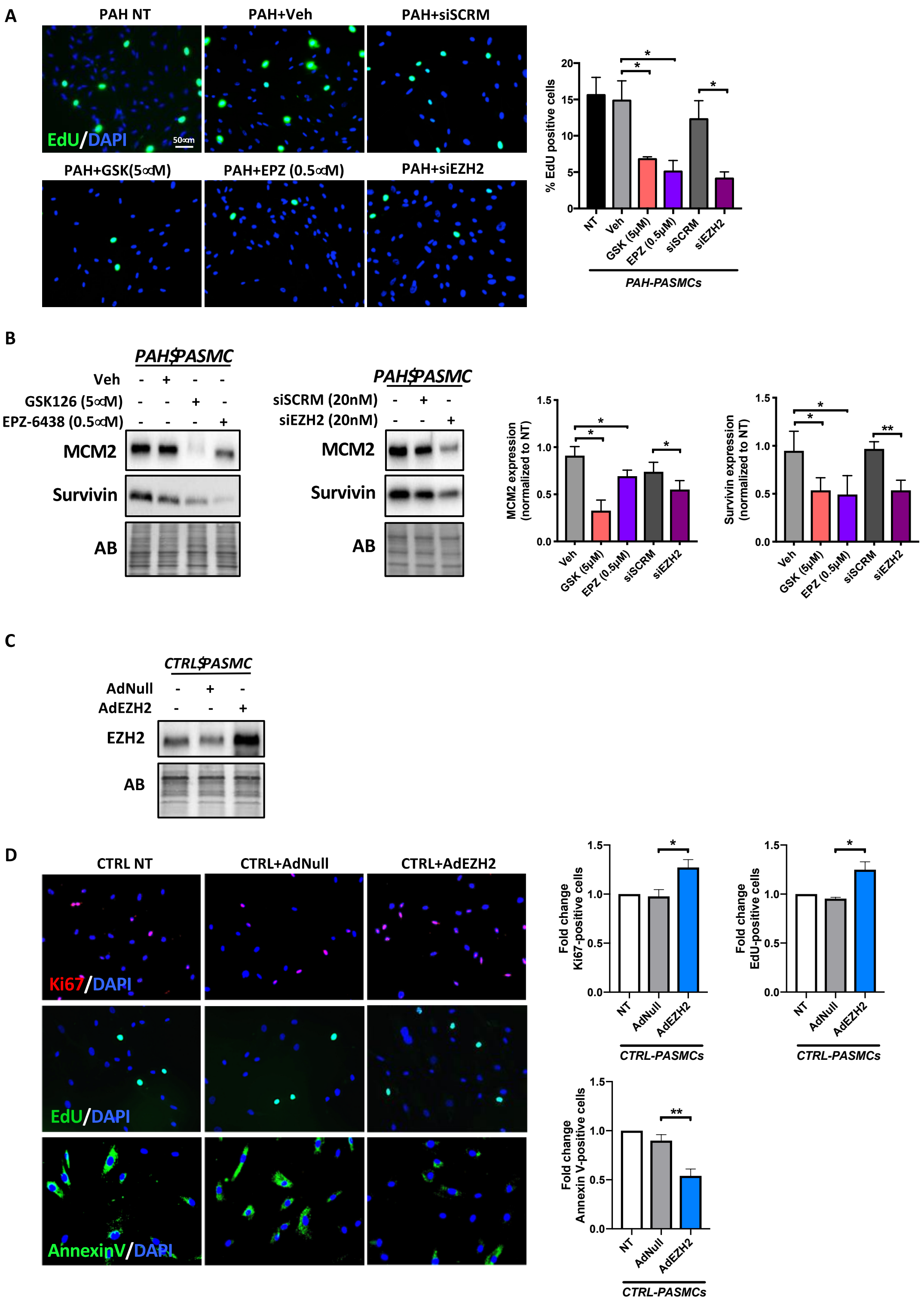

Figure S2. Effects of EZH2 inhibition and overexpression on PASMC proliferation and resistance to apoptosis. A. Proliferation (EdU labeling) was measured in PAH-PASMCs (n=3) treated or not with siEZH2, GSK126 and EPZ-6438 or their respective controls for 48 hours. Graph shows the percentage of cells with positive nuclear EdU staining. Pharmacological or molecular

inhibition of EZH2 reduces PAH-PASMC proliferation. **B**. Representative Western blots and corresponding densitometric analyses of MCM2 and Survivin in PAH-PASMCs (n=3-6) exposed to GSK126, EPZ6438, siEZH2 or their respective controls for 48 hours. Inhibition of EZH2 diminishes MCM2 and Survivin expression levels in PAH-PASMCs. **C**. Representative Western blot of EZH2 in control (CTRL) PASMCs infected or not with an adenovirus encoding human EZH2 (AdEZH2) or with an "empty" adenoviral vector (AdNull). **D**. Representative images and quantitative analysis of Ki67, EdU and Annexin V-positive CTRL-PASMCs (n=4) overexpressing or not EZH2. Forced expression of EZH2 increases CTRL-PASMC proliferation and resistance to apoptosis. Protein expression was normalized to Amido black (AB). Data are presented as mean±SEM; *P<0.05 and **P<0.01.

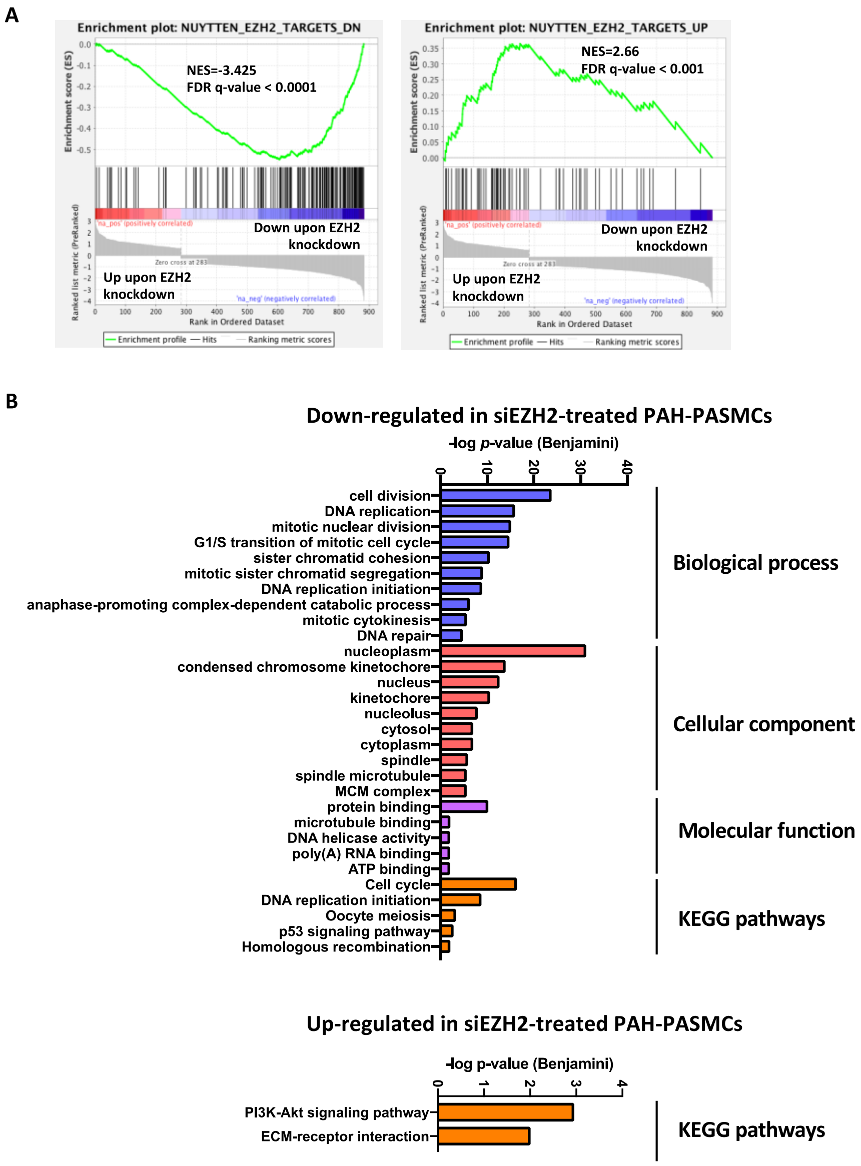

Figure S3. GO and KEGG analysis of differentially expressed genes upon EZH2 knockdown. A. Gene set enrichment analysis (GSEA) charts showing that siEZH2-affected genes in PAH-PASMCs from our RNA-seq data display a significant positive relationship with predefined subset of genes significantly altered in human prostate cancer cells following EZH2 suppression by siRNA. **B.** Gene Ontology (GO) and Kyoto Encyclopedia of Gene and Genomes (KEGG) pathway enrichment results for down- and up-regulated genes between siEZH2- and siSCRM-treated PAH-PASMCs. Blue, red, pink and orange bars indicate biological process, cellular component, molecular function and KEGG pathways, respectively.

A

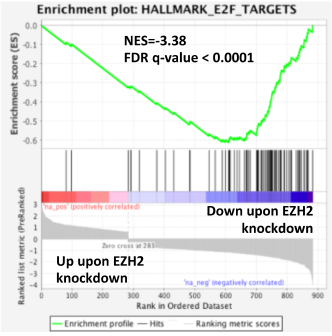

Index	Name	Adjusted p-value	Combined score
1	FOXM1 ENCODE	2.195e-35	1623.48
2	E2F4 ENCODE	2.310e-77	1565.70
3	SIN3A ENCODE	3.647e-22	188.01
4	NFYA ENCODE	3.004e-24	157.43
5	E2F1 CHEA	4.198e-16	133.87
6	NFYB ENCODE	6.252e-22	111.39
7	E2F6 ENCODE	8.536e-20	102.45
8	IRF3 ENCODE	9.203e-10	73.56
9	MAX ENCODE	3.057e-10	51.33
10	SP1 ENCODE	6.136e-7	44.56

B

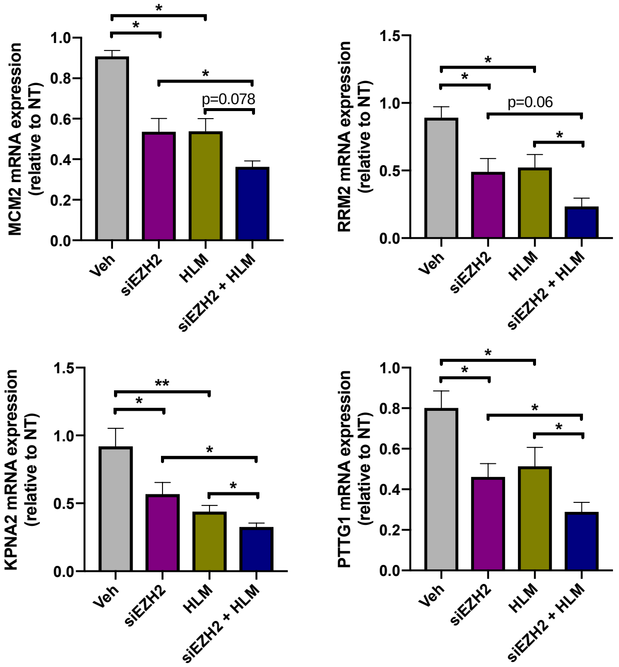

C

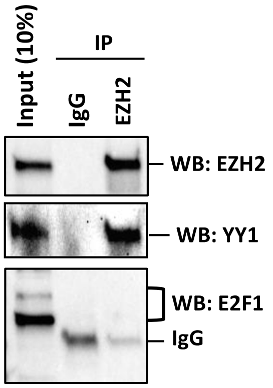

Figure S4. Potential cooperation between EZH2 and E2Fs in stimulation of genes involved in cell proliferation. A. Gene set enrichment analyses using GSEA and Enrichr showing that genes downregulated upon EZH2 silencing are enriched in targets of E2Fs. **B.** Expression levels of MCM2, RRM2, KPNA2 and PTTG1 assessed by real time quantitative PCR in PAH-PASMCs (n=5) treated with siEZH2 and/or HLM006474 (HLM) for 48 hours. **C.** PAH-PASMC cell lysate was pulled down with IgG or EZH2 antibody followed by Western blotting with YY1, a positive control, and E2F1 antibody. Representative immunoblot showing that EZH2 does not interact with E2F1 in PAH-PASMCs. Results are representative of four independent experiments performed on four different PAH-PASMC cell lines. Data are presented as mean±SEM; *P<0.05 and **P<0.01.

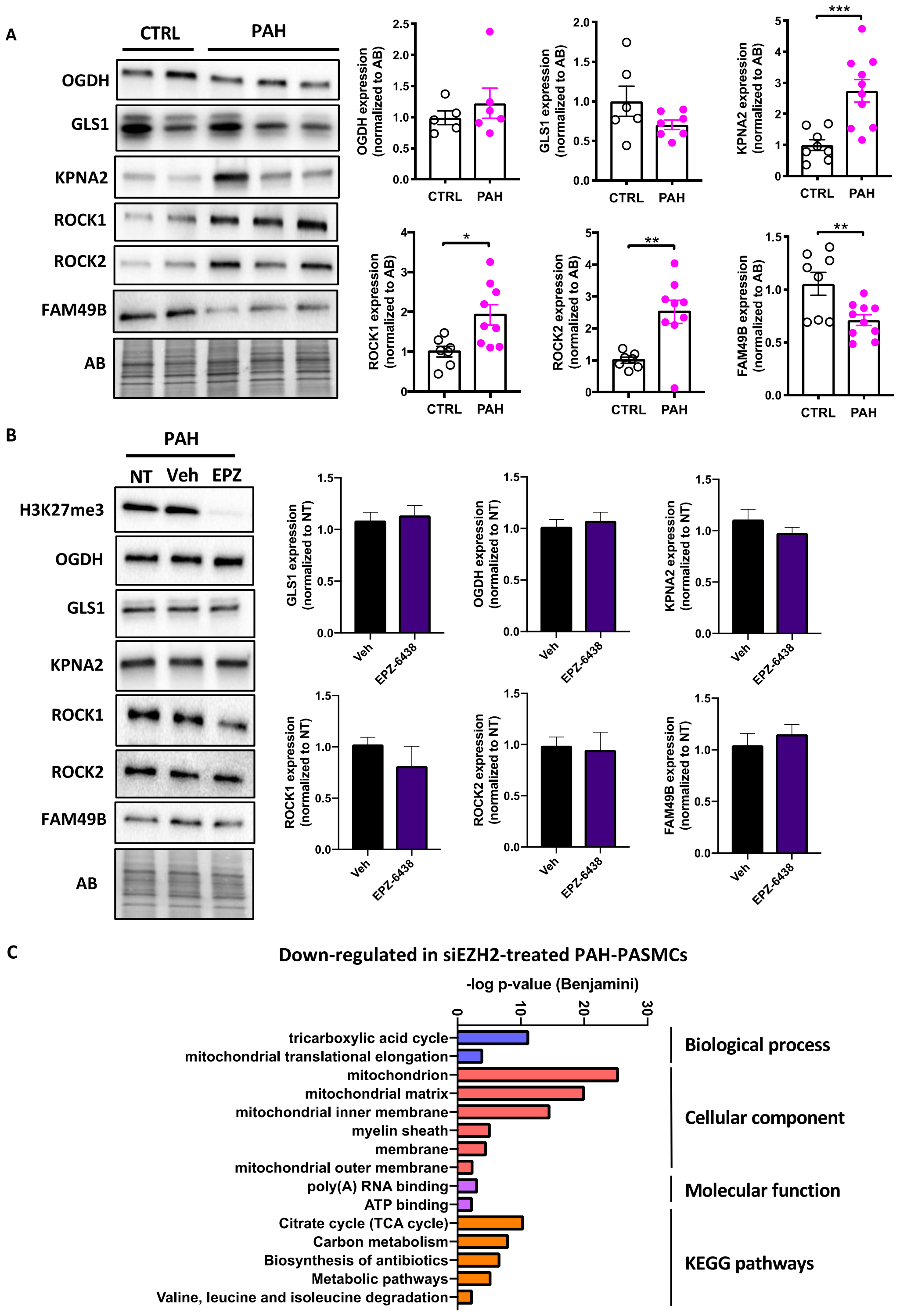

Figure S5. Analysis of protein expression levels of selected DEPs between control and PAH-PASMCs, and effects of pharmacological EZH2 inhibition. A. Representative Western blots and corresponding densitometric analysis of OGDH, GLS1, KPNA2, ROCK1, ROCK2 and FAM49B in PASMCs isolated from control (n=5-8) and PAH (n=6-10) patients. **B.** Representative

Western blots and corresponding densitometric analysis of H3K27me3, OGDH, GLS1, KPNA2, ROCK1, ROCK2 and FAM49B in PAH-PASMCs (n=6) treated or not with EPZ-6438 or its vehicle for 48 hours. **C.** Gene Ontology (GO) and Kyoto Encyclopedia of Gene and Genomes (KEGG) pathway enrichment results for down-regulated proteins between siEZH2- and siSCRM-treated PAH-PASMCs. Blue, red, pink and orange bars indicate biological process, cellular component, molecular function and KEGG pathways, respectively. Protein expression was normalized to Amido black (AB). Data are presented as mean±SEM; *P<0.05; **P<0.01 and ***P<0.001.

	CTL (n=22)	PAH (n=20)
Age (years	45±17	51±15
Gender (female (%))	8 (36.4%)	13 (65%)
PAH group		
HPAH		3 (15%)
IPAH		9 (45%)
SSc-PAH		8 (40%)
Autopsy (n(%))		10 (50%)
Transplantation (n(%))		10 (50%)
Pulmonary hemodynamics		
mPAP (mmHg)		55,6±12
CO (L.min-1)		4.5±1.5
PVR (dyne.sec-1 .cm-5)		744±223
Medication		
Endothelin receptor antagonist		10 (50%)
PDE5 inhibitor		12 (60%)
Prostacyclin analog		6 (30%)
Unknown		2 (10%)

Table S1. Clinical characteristics of PAH patients and controls. Values are means ± SD. HPAH: heritable PAH; IPAH: idiopathic PAH; SSc-PAH: Systemic sclerosis-associated PAH; mPAP: mean pulmonary arterial pressure; CO: cardiac output; PVR: pulmonary vascular resistance; PDE5: phosphodiesterase-5. Note than some patients take more than one type of medication.

		Sexe	Age	PAH	Tissues/Cells used
**CTL**	#1	M	41	-	PASMCs
	#2	M	45	-	PASMCs
	#3	F	17	-	PASMCs
	#4	F	35	-	PASMCs
	#5	M	21	-	PASMCs
	#6	M	43	-	PASMCs
	#7	F	56	-	PASMCs
	#8	M	21	-	PASMCs
	#9	F	32	-	PASMCs
	#10	F	49	-	PA, Lungs
	#11	M	56	-	PA, Lungs
	#12	M	68	-	PA, Lungs
	#13	M	65	-	PA, Lungs
	#14	F	48	-	PA, Lungs
	#15	M	52	-	PA, Lungs
	#16	M	78	-	PA, Lungs
	#17	F	43	-	Lungs
	#18	F	74	-	Lungs
	#19	M	29	-	Lungs
	#20	M	31	-	Lungs
	#21	M	28	-	Lungs
	#22	M	58	-	PASMCs
**PAH**	#1	F	23	iPAH	PASMCs, PA, Lungs
	#2	M	52	iPAH	PASMCs, PA, Lungs
	#3	M	59	iPAH	PASMCs, PA
	#4	M	45	iPAH	PASMCs, Lungs
	#5	F	35	Heritable (BMPR2)	PASMCs
	#6	F	32	Heritable	PASMCs
	#7	M	39	iPAH	PASMCs
	#8	M	65	iPAH	PASMCs, PA
	#9	F	45	SSc-PAH	PASMCs, PA
	#10	F	76	SSc-PAH	PASMCs, PA, Lungs

	#11	M	52	iPAH	PASMCs
	#12	F	61	Heritable (BMPR2)	PA, lungs
	#13	F	72	PAH-SSc	PA, lungs
	#14	F	46	iPAH	PA, lungs
	#15	F	47	PAH-SSc	PA, lungs
	#16	F	53	PAH-SSc	PA, lungs
	#17	F	54	PAH-SSc	PA
	#18	M	77	HTAP-SSc	PA
	#19	F	57	iPAH	Lungs
	#20	F	36	PAH-SSc	Lungs

Table S2. Detailed tissue characteristics used in this study.

Gene	Sequence (5'-3')	Amplicon size (bp)	Exon location
RRM2	F: GGT TCT TTT GCG TCG ATA TTC TG<br>R: GGT TTG TGT ACC AGG TGT TTG	140	7-8
PTTG1	F: GAT GAA TGC GGC TGT TAA GAC<br>R: CTT CAG CCC ATC CTT AGC AA	111	1-2
MCM2	F: CAC CGC TTC AAG AAC TTC CT<br>R: TGC CAA GTC CTC ATA GTT CAC	126	4-5
KPNA2	F: AAG GAT GAC CAG ATG CTG AAG<br>R: ATC AAC AGA CCA ATT TAC AGT GC	111	3-4
18S	F: CGC ACG GCC GGT ACA GTG AA<br>R: GGG AGA GGA GCG AGC GAC CA	76	1

Table S3. List of primer sequences.

Antigen	Antibody/clone	Source	Application	Dilution
EZH2	Rabbit monoclonal (D2C9)	Cell Signaling (#5246)	WB<br>IF	1:1000<br>1:100
αSMA	Mouse monoclonal (1A4)	Sigma-Aldrich (#A2547)	IF	1:200
H3K27me3	Rabbit monoclonal (C36B1)	Cell Signaling (#9733)	WB	1:1000
MCM2	Rabbit polyclonal	Bethyl Laboratories (A300-191A)	WB	1:1000
Survivin	Rabbit monoclonal (71G4B7)	Cell Signaling (#2808)	WB	1:1000
E2F1	Mouse monoclonal (KH95)	Invitrogen (#32-1400)	WB	1:1000
YY1	Mouse monoclonal (H-10)	Santa Cruz Biotechnology (sc-7341)	WB	1:500
OGDH	Rabbit polyclonal	Proteintech (15212-1)	WB	1:5000
GLS1	Mouse monoclonal (3A12A1)	Proteintech (66265-1-Ig)	WB	1:1000
ROCK1	Mouse monoclonal (46)	BD Biosciences (#611137)	WB	1:500
ROCK2	Rabbit polyclonal	Bethyl Laboratories (A300-047A)	WB	1:1000
KPNA2	Rabbit polyclonal	Abcam (ab70150)	WB	
FAM49B	Mouse monoclonal (D-8)	Santa Cruz Biotechnology (sc-390478)	WB	1:1000
Ki67	Rabbit polyclonal	Millipore (AB9260)	IF	1:300

Table S4. List of primary antibodies used for IF, WB and Co-IP
